# Reply to Pallotti et al. Comment on “Boitrelle et al. The Sixth Edition of the WHO Manual for Human Semen Analysis: A Critical Review and SWOT Analysis. *Life* 2021, *11*, 1368”

**DOI:** 10.3390/life12071046

**Published:** 2022-07-13

**Authors:** Florence Boitrelle, Rupin Shah, Ramadan Saleh, Ralf Henkel, Hussein Kandil, Eric Chung, Paraskevi Vogiatzi, Armand Zini, Mohamed Arafa, Ashok Agarwal

**Affiliations:** 1 Reproductive Biology, Fertility Preservation, Andrology, CECOS, Poissy Hospital, 78300 Poissy, France; florenceboitrelle@yahoo.fr; 2 UVSQ, INRAE, BREED, Paris Saclay University, 78350 Jouy-en-Josas, France; 3 Division of Andrology, Department of Urology, Lilavati Hospital and Research Centre, Mumbai 400050, India; rupinurvashishah@gmail.com; 4 Department of Dermatology, Venereology and Andrology, Faculty of Medicine, Sohag University, Sohag 82524, Egypt; salehr2010@yahoo.com; 5 Ajyal IVF Center, Ajyal Hospital, Sohag 82524, Egypt; 6 Department of Metabolism, Digestion and Reproduction, Imperial College London, London SW7 2AZ, UK; ralf.henkel@logixxpharma.com; 7 Department of Medical Bioscience, University of the Western Cape, Bellville 7493, South Africa; 8 American Center for Reproductive Medicine, Cleveland Clinic, Cleveland, OH 44195, USA; 9 LogixX Pharma, Theale, Reading, Berkshire RG7 4AB, UK; 10 Fakih IVF Fertility Center, Abu Dhabi 31452, United Arab Emirates; hkandil@gmail.com; 11 AndroUrology Centre, Brisbane, QLD 4230, Australia; ericchg@hotmail.com; 12 Department of Urology, Princess Alexandra Hospital, University of Queensland, Brisbane, QLD 4120, Australia; 13 Andromed Health & Reproduction, Fertility Diagnostics Laboratory, Maroussi, 15126 Athens, Greece; evivogiatzi@gmail.com; 14 Department of Surgery, McGill University, Montreal, QC H3A 1G5, Canada; ziniarmand@yahoo.com; 15 Department Andrology Department, Cairo University, Giza 11562, Egypt; mohamedmostafaarafa@gmail.com; 16 Urology Department, Hamad Medical Corporation, Doha P.O. Box 3050, Qatar; 17 Global Andrology Forum, Moreland Hills, OH 44022, USA

We would like to thank F. Pallotti and his colleagues for their positive comments [1] on our SWOT analysis of the strengths, weaknesses, threats and opportunities of the sixth edition of the WHO manual on semen analysis [2]. They have raised two concerns: the re-introduction of the category of rapidly moving sperm, and the dropping of the reference range.

With regard to the re-introduction of the distinction between rapidly progressive (type a) motility and sluggish progressive (type b) motility, Pallotti et al. raised the point that this distinction is difficult to make visually and will, therefore, lead to “approximation” and subjective reporting with “reduced standardization”. This is a valid concern and was the reason why the editors of the fifth edition of the WHO manual removed this distinction (which was present in the fourth edition) and created the combined category of “progressive motility”. However, this move received considerable criticism, and hence, after a review of available evidence, the editors of the sixth edition concluded that differentiation between rapid and sluggish motility was clinically relevant and of prognostic significance, and re-introduced this categorization while acknowledging the difficulty that this may pose in the laboratory. Obviously, as we highlighted, new studies are needed. If the utility of identifying the presence of “rapid progressive” sperm is validated, then future editions of the manual may have to recommend the incorporation of artificial intelligence-based (AI-based) optical systems already available today to assess motility as part of the standard semen analysis.

Secondly, the dropping of the reference range is indeed the most important change in the sixth edition. We agree with Pallotti et al. that the dropping of reference values will not necessarily be problematic for the seasoned male infertility clinician who is aware of the limitations of sperm analysis in the infertility work-up. In fact, this frees the clinicians to use their judgement about who needs treatment without being constrained by a cut-off that may incorrectly label a male as fertile. However, according to the latest EAU recommendations [3], basic semen analysis has been placed at the forefront of the infertile man’s clinical evaluation and is part of the initial management of the infertile couple.

Hence, often it will be the primary care physician (the couple’s gynecologist who is usually not specialized in assisted reproduction or andrology) who will interpret the semen report and, in the absence of a “standard” or “reference range”, may find it difficult to decide whether to refer the man for specialized management. The sixth edition has suggested that this problem may be resolved by creating “decision limits” instead. However, this was not elucidated further and is a work-in-progress that should be resolved in the next edition of the manual.

Thus, currently, the elimination of reference limits, without providing an alternative for interpretation of the semen analysis, makes the sixth edition an excellent technical manual, but it limits its role as a clinical guide.
